# Plasma metabolites in childhood Burkitt lymphoma cases and cancer-free controls in Uganda

**DOI:** 10.1007/s11306-024-02130-1

**Published:** 2024-06-28

**Authors:** Jiaqi Huang, Hadijah Nabalende, M. Constanza Camargo, Jacqueline Lovett, Isaac Otim, Ismail D. Legason, Martin D. Ogwang, Patrick Kerchan, Tobias Kinyera, Leona W. Ayers, Kishor Bhatia, James J. Goedert, Steven J. Reynolds, Peter D. Crompton, Steven C. Moore, Ruin Moaddel, Demetrius Albanes, Sam M. Mbulaiteye

**Affiliations:** 1 National Clinical Research Center for Metabolic Diseases, Metabolic Syndrome Research Center, Key Laboratory of Diabetes Immunology, Department of Metabolism and Endocrinology, Ministry of Education, The Second Xiangya Hospital of Central South University, Changsha, Hunan 410011 China; 2 Xiangya School of Public Health, Central South University, Changsha, Hunan 410128 China; 3 CSU-Sinocare Research Center for Nutrition and Metabolic Health, Changsha, China; 4 EMBLEM Study, St. Mary’s Hospital, Lacor, Gulu & African Field Epidemiology Network, Kampala, Uganda; 5 Division of Cancer Epidemiology and Genetics, National Cancer Institute, National Institutes of Health, HHS,, 9609 Medical Center Dr, Rm. 6E-118, MSC 3330, Bethesda, MD 20892 USA; 6 Laboratory of Clinical Investigation, Intramural Research Program, National Institute on Aging, NIH, Baltimore, MD USA; 7 EMBLEM Study, Arua & African Field Epidemiology Network, Kuluva Hospital, Kuluva, Kampala, Uganda; 8 Department of Pathology, The Ohio State University, Columbus, OH USA; 9 Division of Intramural Research, National Institute of Allergy and Infectious Diseases, National Institutes of Health, Bethesda, Maryland USA

**Keywords:** Burkitt lymphoma, Non-hodgkin lymphoma, Epidemiology, Epstein-Barr virus, *Plasmodium Falciparum* malaria, Metabolomics

## Abstract

**Introduction:**

Burkitt lymphoma (BL) is an aggressive non-Hodgkin lymphoma associated with *Plasmodium falciparum* and Epstein-Barr virus, both of which affect metabolic pathways. The metabolomic patterns of BL is unknown.

**Materials and methods:**

We measured 627 metabolites in pre-chemotherapy treatment plasma samples from 25 male children (6–11 years) with BL and 25 cancer-free area- and age-frequency-matched male controls from the Epidemiology of Burkitt Lymphoma in East African Children and Minors study in Uganda using liquid chromatography-tandem mass spectrometry. Unconditional, age-adjusted logistic regression analysis was used to estimate odds ratios (ORs) and their 95% confidence intervals (CIs) for the BL association with 1-standard deviation increase in the log-metabolite concentration, adjusting for multiple comparisons using false discovery rate (FDR) thresholds and Bonferroni correction.

**Results:**

Compared to controls, levels for 42 metabolite concentrations differed in BL cases (FDR < 0.001), including triacylglyceride (18:0_38:6), alpha-aminobutyric acid (AABA), ceramide (d18:1/20:0), phosphatidylcholine ae C40:6 and phosphatidylcholine C38:6 as the top signals associated with BL (ORs = 6.9 to 14.7, *P* < 2.4✕10^− 4^). Two metabolites (triacylglyceride (18:0_38:6) and AABA) selected using stepwise logistic regression discriminated BL cases from controls with an area under the curve of 0.97 (95% CI: 0.94, 1.00).

**Conclusion:**

Our findings warrant further examination of plasma metabolites as potential biomarkers for BL risk/diagnosis.

**Supplementary Information:**

The online version contains supplementary material available at 10.1007/s11306-024-02130-1.

## Introduction

Burkitt lymphoma (BL) is an aggressive non-Hodgkin lymphoma that represents a significant health problem in African children (López et al., 2022). The risk factors of BL in sub-Saharan Africa (SSA) include *Plasmodium falciparum* (the parasite that causes malaria) (Derkach et al., 2019) and Epstein-Barr virus (EBV) (Bornkamm, 2009). These infections may influence BL risk by triggering the hallmark translocation of *c-MYC* into the vicinity of immunoglobulin genes on chromosomes 14, 2, or 22 (*IG*∷*MYC*)(Alaggio et al., 2022).

BL can be treated successfully in high-income countries (Ozuah et al., 2020), however, cure rates are low in SSA because of limited diagnostic capacity (Chamba et al., 2023), which leads to significant delay and often compels local clinicians to base treatment decisions on clinical diagnosis. The association of BL with *P. falciparum* and EBV holds promise for innovative, potentially less invasive, faster point-of-care diagnosis of BL, but few studies have evaluated malaria markers, with most focusing on EBV plasma markers (Coghill et al., 2020; Donati et al., 2006; Westmoreland et al., 2017; Xian et al., 2021). For example, in one study that evaluated serological markers for EBV infection, immunoglobulin G antibodies against four EBV peptides (BHRF1, BMRF1, BBLF1, and BZLF1) were associated with moderate discrimination of BL cases from controls, based on an area under the curve (AUC) of 0.76 (95% confidence interval: 0.6 to 0.9) (Coghill et al., 2020). Three studies of EBV DNA in plasma reported better discrimination of BL cases from controls using study-defined EBV DNA thresholds (Donati et al., 2006; Westmoreland et al., 2017; Xian et al., 2021) of which one achieved an AUC of 0.94 (95% CI: 0·85–1·00) in a study of 25 Ugandan children with BL compared to 25 controls (Xian et al., 2021).

Metabolomics offer a powerful platform for investigating biology and biomarkers of risk for multiple conditions (Andrade et al., 2020; Moaddel et al., 2022), yet, to date only limited metabolomic data are available in relation to the risk of BL in humans. In mice, measured serum metabolites discriminate mice implanted with Raji cells (a BL-derived cee line) from control wild-type mice based on differential abundance of metabolites (Yang et al., 2017). Altered metabolite profiles, notably, differences in metabolites in the glycolytic (Na et al., 2019), cholesterol, and lipid pathways (Abdrabou et al., 2021), have been used to demonstrate differences in patients with severe malaria as well as those with asymptomatic infection with low parasitemia (< 1000 parasites/µL) versus those with undetectable parasitemia (Faucher et al., 2002). These associations may have possible relevance for BL in which alterations in lipid metabolism have been reported (Ambrosio et al., 2012) and might be correlated with EBV-mediated effects (Li et al., 2004).

Here, we carried out a pilot study where we measured plasma metabolites in children from Uganda with and without BL (Peprah et al., 2020) in order to obtain their baseline metabolomic profiles, to assess associations with BL, given that both malaria and EBV, which are established environmental and endogenous risk factors of BL, have been associated with disruptions in metabolite levels. We aimed to examine the potential diagnostic utility of blood-based metabolomics for childhood BL.

## Methods

### Study population

For the present investigation, we selected specimens from 25 male cases and 25 male controls aged 6–11 years enrolled from two districts in Uganda as part of the Epidemiology of Burkitt lymphoma in East African children and minors (EMBLEM) study (Peprah et al., 2020; Legason et al., 2017). Blood samples from BL cases were collected prior to any chemotherapy (including steroids) received in the hospital. BL cases confirmed by local histology/cytology (61% of cases), otherwise by clinical features, imaging, and laboratory results, were enrolled as previously described (Peprah et al., 2020). Controls were enrolled from 100 random representative villages selected in the study area in Uganda, with frequency matching to the age and sex distribution of historical cases in the area (Maziarz et al., 2017). The controls were healthy children based on the fact they were enrolled in their homes at a time where they were living normal lives without symptoms of BL, no history of any cancer diagnosis, and had no complaints requiring medical care. So, the controls were assumed to be cancer free individuals. Venous blood specimens were collected in EDTA from children diagnosed with BL (Peprah et al., 2020) for clinical tests and research. Research blood samples were separated into plasma, buffy coat, and red blood cell fractions and stored frozen at -80^o^ C until testing (Maziarz et al., 2017). *P. falciparum* infection was determined using microscopy of thick-film blood smears or rapid diagnostic tests (RDT) to detect the *P. falciparum*-specific malaria histidine-rich protein 2 (*Pf*-HRP2) and the pan-lactate dehydrogenase (pLDH) antigen shared by other Plasmodia that parasitize humans. The weighted *P. falciparum* prevalence (*Pf*PR) in these districts was estimated to vary from 55 to 75% (Maziarz et al., 2017) and the average annual cumulative *P. falciparum* infections per child in the study regions was estimated using Malaria Atlas Project data (Weiss et al., 2019) to vary from 300 to 400 (Broen et al., 2023). The prevalence of stool parasitic infection, evaluated through the Kato method, was minimal (< 1–2%), thus it was excluded from the analytical consideration of this comorbidity.

### Determination of plasma metabolite concentrations

Aliquots (50 µl) of previously unthawed plasma, from samples collected between 8:00–11:00 AM before breakfast, to minimize post-prandial distortions of circulating metabolites, were retrieved and shipped on dry ice for analysis. Metabolites were extracted from plasma (10 µl) and concentrations obtained using the MxP 500 (Biocrates Life Science AG, Austria) following the manufacturer’s protocol. Metabolites were measured using a Nexera High Performance Liquid Chromatography (HPLC) system (Shimadzu) coupled to a 6500 QTRAP® mass spectrometer (AB Sciex) with an electrospray ionization source as previously described (Moaddel et al., 2022). Samples were analyzed by flow injection analysis-tandem mass spectrometry (FIA-MS/MS) and liquid chromatography-tandem mass spectrometry (LC-MS/MS) using a 6500 + QTRAP® instrument. Briefly, two UHPLC methods were run using the MxP Quant 500 Column System for the LC-MS/MS methods with solvent A as water containing 0.2% formic acid) and solvent B as acetonitrile containing 0.2% formic acid. Analytes in the LC-MS/MS part are quantified using either external 7-point calibration curves with labeled standards or internally with labeled standards. Zero samples were used for analyte LODs for LC-MS/MS. For the FIA-MS/MS method, the FIA plate was run at a flow rate of 30 µL/min with FIA solvent as the mobile phase. Analytes in the FIA-MS/MS part are quantified using internal standards. Zero samples were used for analyte LODs for FIA Method 1 and MetIDQ was used to provide LODs for FIA method 2. Concentrations were calculated using the Analyst/MetIDQ software and reported in µmol/L. Data were quantified using appropriate mass spectrometry software (Sciex Analyst®) and imported into Biocrates MetIDQ™ software for further analysis. The data were normalized to internal quality controls. Laboratory staff were blinded to case-control status of the samples. In the quality control, the coefficients of variations (CVs) were < 10% for the majority of the measured metabolites and < 20% for the measured lipids, with few exceptions; 15 metabolites had CV > 30%, none of which were associated with BL in our study.

### Statistical analysis

We analyzed metabolites with values *≥* the limit of detection (LOD) in at least 24% (> 12) of the subjects tested, resulting in 187 metabolites being excluded (Supplementary Tables 1 and 2) and 440 metabolites being studied (Supplementary Table 3). For these 440 metabolites, we set values that were below the LOD (“as missing values”) to one half the value of LOD of that metabolite. Results were log-transformed for analysis.

Unconditional logistic regression models were fit to estimate odds ratios (ORs) and 95% confidence intervals (95% CIs) for the association between BL and one standard deviation (SD) of the log-transformed value of the plasma metabolite. Primary models were adjusted for age at enrollment in single-year increments. Age is positively correlated with cumulative exposure to *P. falciparum* and naturally acquired immunity. Models were further adjusted for *P. falciparum* infection as a factor (negative or positive). Sensitivity analyses were performed that stratified by median age at enrollment, *P. falciparum* infection (negative and positive) and median hemoglobin concetration to assess effect modification of the primary association. Given the small sample size, we calculated the false-discovery rate (FDR) to identify potentially important patterns (FDR ≤ 0.001). We performed forward stepwise logistic regression models to select a subset of metabolites that were independently associated with BL using a *p* *≤* 0·0003 (top 12 metabolites) criterion to enter into the model and a *p* < 0.05 to remain in the model. We assessed the performance of malaria-related variables (age, *P. falciparum* infection, and hemoglobin concentration) versus models also including the metabolites identified in the stepwise models to discriminate between children with BL versus those without BL using the Receiver Operating Curve (ROC), with the AUC a as summary performance measure.

Associations between BL and biochemical pathways for 21 metabolite-sub-classes that together comprise the 440 metabolites were assessed. We calculated *p*-values of association for each pathway using a parametric bootstrap method, such that within each bootstrap replication, *p*-values were generated from a vector of score test statistics from an estimated covariance matrix with a multivariate normal distribution (mean = 0), based on 100,000 permutations. We also performed a principal components analysis (PCA) using the varimax rotation approach. The pathway score by the first generated principal component for each class was used as a predictor in unconditional logistic regression to assess the associations between BL and the obtained pathway score, controlling for age at enrollment, *P. falciparum* infection and hemoglobin concetration . Pathway associations were subjected to Bonferroni correction based on 21 chemical class tests performed, i.e., *P* = 0.0024 (0·05/21). Finally, to improve the interpretation of our metabolite data, we performed exploratory analysis using calculated custom sums and ratios based on predefined values trademarked by Biocrates (MetaborIndicator™) to assess biological pathways (e.g., inflammation) and then examined the association between BL and the custom sums and ratios in the 440 metabolites. The ratio analysis was not adjusted for multiple comparisons.

All statistical analyses were performed using SAS version 9·4 (SAS Institute, Cary, NC), and R version 3·6 ·1 (R Development Core Team, Vienna, Austria).

## Results

We studied 25 children with BL and 25 children without BL (Table 1). The mean age of the participants with recorded age (*n* = 46) was 8 years; the exact age was not known for four children. There were no differences between cases and controls with respect to height, weight, or body mass index (Table 1). *P. falciparum* infection was detected in seven (28%) cases and 11 (44%) controls (*P* = 0·38), but none of the participants had malaria symptoms. Mean hemoglobin concentration was lower in BL cases than cancer-free controls (10.0 versus 12·4 g/dL, *P* < 0·0001), consistent with the notion that BL cases are exposed to more malaria and are therefore more likely to have mild anemia (defined as < 11 g/dL) (White, 2018).

**Table 1:** Characteristics of Burkitt lymphoma cases and cancer-free controls in the study^1^

Characteristics	BL cases (*n* = 25)	Controls (*n* = 25)	*P*-value^2^
Age at enrollment (SD), years	8·1 (1·8)	8·0 (1·7)	0·67
*P. falciparum* infection (parasitemia/antigenemia)			0·38
Negative	18 (72%)	14 (56%)	
Positive	7 (28%)	11 (44%)	
Hemoglobin concentration, g/dL^3^	10·0 (1.85)	12·4 (0·740)	< 0.001
Height, (cm)^4^	123.2 (7.1)	126.8 (0.7)	0.178
Weight (kg) ^4^	22.8 (2.8)	24.9 (5.5)	0.106
Body mass index (kg/m^2^)^4^	15.0 (1.3)	15.3 (1.4)	0·495

^1^Values are means (SD: standard deviations) unless otherwise indicated

^2^*P* values are derived from *t test* for continuous variables, and from Fisher’s Exact test for the categorical variables

^3^Values missing for one cancer-free control and for 3 BL cases

^4^Values missing for 2 subjects, both BL cases

**Fig. 1:**
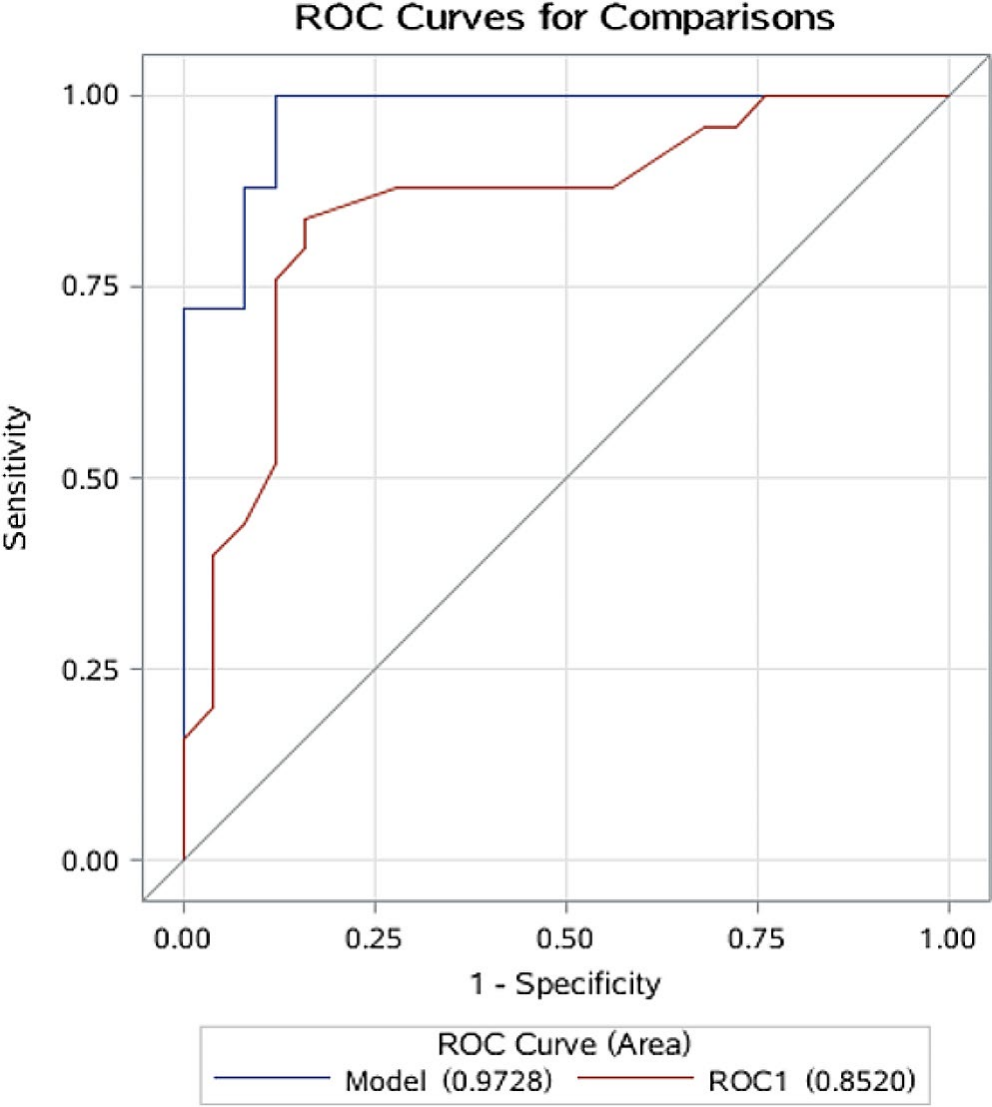
Receiver operating curve (ROC) and area under the curve (AUC) as a summary of performance of two metabolites identified in stepwise models plus malaria variables (age, *P. falciparum* status, and hemoglobin conetration ) or when using the malaria variables alone to discriminate Burkitt Lymphoma cases from controls Results for red ROC curve: AUC = 0·85 (95% CI: 0·74, 0·96) for risk factors including age at enrollment, *P. falciparum* status and hemoglobin concetration  (low versus high). Results for the blue curve: AUC = 0·97 (95% CI: 0·94, 1·00) for the above-mentioned risk factors and two metabolites selected from the forward stepwise regression models, including triacylglyceride (18:0_38:6) and AABA (alpha-aminobutyric acid) (AUC 0·58 versus 0·97, *P* value for difference = 0·043)

The primary analysis identified 42 plasma metabolites associated with BL, including three amino acids and 39 lipids (Table 2), based on an FDR ≤ 0·001 (Supplementary Tables 4 and 5). Most of these metabolites (*n* = 41) were associated with elevated ORs for BL (4·47 − 25·2) and included triglycerides (TAG), phosphatidylcholines (PCs), diglycerides, hexosylceramides, amino acids and biogenic amines (Table 2). The TAGs were generally positively associated with BL status (Table 2). Putrescine was the only a biogenic amine associated with BL, and only one metabolite, homoarginine, was associated with decreased ORs for BL (OR = 0.1, 95% CI: 0.03, 0.35) (Table 2). Five metabolites, including TAG (18:0_38:6), alpha-aminobutyric acid (AABA), ceramide (d18:1/20:0), PC ae C40:6 and PC ae C38:6, were top signals associated with BL status (ORs = 6.9 to 14.7, *P* < 2.4✕10^− 4^). These associations were not changed in sensitivity analyses adjusting for asymptomatic *P. falciparum* infection (Table 2). There were no material differences in the estimates of associations across subgroups based on median age at enrollment, *P. falciparum* infection (negative and positive) and median hemoglobin concentration, but the small sample size led to high confidence intervals in the subgroups. Two metabolites associated with elevated ORs of BL, namely, TAG (18:0_38:6) and AABA, were selected using stepwise logistic regression. Models that included these two metabolites, baseline age, *P. falciparum* infection and hemoglobin concentration discriminated BL cases from controls with an AUC of 0·97 (97% CI: 0·94, 1·00). This model was a significant improvement over the model that only included *P. falciparum* infection, baseline age, and hemoglobin level (AUC = 0·85, 95% CI: 0·74, 0·96; P for difference = 0·043, Fig. 1).

**Table 2:** Odds ratios and 95% CIs (per 1-SD) for the association of endemic Burkitt lymphoma and plasma metabolites reaching the FDR < 0.001 threshold based on 25 case-cancer-free control pairs

Metabolite^a, b^	OR (95% CI)^c^	*P*	OR (95% CI)^d^	*P*
Amino Acid Related				
Alpha-aminobutyric acid	11.4 (3.2, 40.2)	1.6 × 10^− 4^	11.2 (3.2, 39.8)	1.9 × 10^− 4^
Homoarginine	0.1 (0, 0.4)	3.5 × 10^− 4^	0.1 (0, 0.3)	3.3 × 10^− 4^
Biogenic Amines				
Putrescine	11.5 (3.0, 44.7)	4.2 × 10^− 4^	12.4 (3.0, 50.7)	4.8 × 10^− 4^
Ceramides				
Ceramide (d18:1/20:0)	6.9 (2.5, 19.1)	2.3 × 10^− 4^	7.4 (2.5, 21.7)	2.5 × 10^− 4^
Ceramide (d18:1/24:1)	6.4 (2.3, 18.3)	4.9 × 10^− 4^	6.2 (2.2, 17.9)	6.8 × 10^− 4^
Ceramide (d18:1/22:0)	5.8 (2.2, 15.2)	3.7 × 10^− 4^	6.0 (2.2, 16.2)	4.5 × 10^− 4^
Diglycerides				
Diacylglyceride (18:1_18:2)	10.3 (2.9, 36.7)	3.4 × 10^− 4^	11.3 (2.8, 45.0)	6.1 × 10^− 4^
Hexosylceramides				
Hexosylceramide (d18:1/16:0)	5.7 (2.2, 14.6)	3.5 × 10^− 4^	5.4 (2.1, 13.9)	4.6 × 10^− 4^
Phosphatidylcholines				
Phosphatidylcholine ae C38:5	25.2 (4.4, 144.8)	3.1 × 10^− 4^	24.1 (4.2, 137.5)	3.5 × 10^− 4^
Phosphatidylcholine ae C36:4	23.3 (4.2, 129.3)	3.2 × 10^− 4^	22.6 (4.1, 126.5)	3.8 × 10^− 4^
Phosphatidylcholine ae C38:6	14.7 (3.5, 61.9)	2.4 × 10^− 4^	14.8 (3.4, 64.5)	3.3 × 10^− 4^
Phosphatidylcholine ae C38:4	11.6 (3.1, 43.5)	2.7 × 10^− 4^	11.4 (2.9, 43.9)	4.3 × 10^− 4^
Phosphatidylcholine ae C40:6	11.3 (3.1, 41.1)	2.4 × 10^− 4^	10.9 (3, 40.3)	3.2 × 10^− 4^
Phosphatidylcholine aa C38:0	7.8 (2.5, 24.6)	4.1 × 10^− 4^	7.5 (2.4, 23.4)	4.9 × 10^− 4^
Phosphatidylcholine aa C32:0	7.6 (2.4, 23.4)	4.6 × 10^− 4^	7.2 (2.3, 22.3)	6.4 × 10^− 4^
Phosphatidylcholine ae C34:0	5.8 (2.2, 15.8)	5.1 × 10^− 4^	5.8 (2.1, 15.8)	6.8 × 10^− 4^
Triglycerides				
Triacylglyceride (18:2_36:0)	16.1 (3.6, 71.5)	2.7 × 10^− 4^	16.4 (3.6, 74.1)	2.7 × 10^− 4^
Triacylglyceride (20:0_32:4)	14.9 (3.4, 64.5)	3.2 × 10^− 4^	25.3 (3.8, 167.3)	8.1 × 10^− 4^
Triacylglyceride (20:4_36:2)	13.7 (3.1, 60.3)	5.7 × 10^− 4^	13.6 (3.1, 60.5)	6.0 × 10^− 4^
Triacylglyceride (18:0_38:6)	10.9 (3.2, 37.2)	1.4 × 10^− 4^	10.9 (3.2, 37.6)	1.5 × 10^− 4^
Triacylglyceride (22:6_34:2)	10.4 (2.9, 37.5)	3.5 × 10^− 4^	10.3 (2.8, 37.5)	4.0 × 10^− 4^
Triacylglyceride (20:4_34:1)	9.9 (2.8, 35.1)	3.9 × 10^− 4^	9.8 (2.7, 34.8)	4.4 × 10^− 4^
Triacylglyceride (20:4_36:3)	9.0 (2.7, 30.3)	4.1 × 10^− 4^	9.3 (2.6, 33.5)	6.3 × 10^− 4^
Triacylglyceride (18:0_36:3)	8.9 (2.8, 28.7)	2.6 × 10^− 4^	9.0 (2.7, 30.2)	3.4 × 10^− 4^
Triacylglyceride (18:2_36:1)	8.7 (2.6, 28.3)	3.7 × 10^− 4^	8.5 (2.6, 28.1)	4.6 × 10^− 4^
Triacylglyceride (18:1_38:5)	8.5 (2.6, 28.0)	4.1 × 10^− 4^	8.5 (2.6, 28.3)	4.7 × 10^− 4^
Triacylglyceride (18:0_36:4)	8.1 (2.5, 26.4)	4.8 × 10^− 4^	8.5 (2.5, 29.3)	7.4 × 10^− 4^
Triacylglyceride (16:0_40:6)	7.5 (2.6, 22.3)	2.6 × 10^− 4^	7.5 (2.5, 22.2)	3.2 × 10^− 4^
Triacylglyceride (22:5_34:1)	7.5 (2.5, 22.1)	2.7 × 10^− 4^	7.5 (2.5, 22.3)	3.2 × 10^− 4^
Triacylglyceride (22:5_34:2)	7.2 (2.5, 21.0)	3.0 × 10^− 4^	7.1 (2.4, 20.9)	3.6 × 10^− 4^
Triacylglyceride (18:2_34:0)	7.2 (2.5, 21.0)	3.1 × 10^− 4^	7.1 (2.4, 20.9)	3.9 × 10^− 4^
Triacylglyceride (16:0_38:6)	6.8 (2.3, 20.3)	5.8 × 10^− 4^	6.7 (2.2, 20.1)	7.2 × 10^− 4^
Triacylglyceride (18:2_38:4)	6.8 (2.4, 18.9)	2.5 × 10^− 4^	6.7 (2.4, 19.0)	3.2 × 10^− 4^
Triacylglyceride (18:1_38:6)	6.7 (2.3, 19.4)	5.0 × 10^− 4^	6.6 (2.2, 19.3)	6.0 × 10^− 4^
Triacylglyceride (20:0_32:3)	6.6 (2.4, 18.7)	3.4 × 10^− 4^	7.1 (2.3, 21.5)	5.3 × 10^− 4^
Triacylglyceride (18:2_32:0)	6.4 (2.3, 18.0)	3.9 × 10^− 4^	6.3 (2.2, 17.8)	4.9 × 10^− 4^
Triacylglyceride (22:6_34:1)	6.0 (2.2, 16.0)	4.1 × 10^− 4^	5.9 (2.2, 16.0)	4.9 × 10^− 4^
Triacylglyceride (16:0_36:4)	5.8 (2.1, 15.6)	5.4 × 10^− 4^	5.6 (2.1, 15.3)	7.5 × 10^− 4^
Triacylglyceride (20:4_36:4)	5.7 (2.1, 15.2)	5.2 × 10^− 4^	5.6 (2.0, 15.4)	8.7 × 10^− 4^
Triacylglyceride (18:2_38:5)	5.3 (2.1, 13.5)	4.3 × 10^− 4^	5.2 (2.0, 13.5)	6.1 × 10^− 4^
Triacylglyceride (18:2_34:1)	4.6 (2.0, 10.8)	3.9 × 10^− 4^	4.5 (1.9, 10.5)	5.2 × 10^− 4^
Triacylglyceride (16:0_36:3)	4.5 (1.9, 10.3)	4.5 × 10^− 4^	4.3 (1.9, 10.0)	6.0 × 10^− 4^

Abbreviations: CI = confidence interval, OR = odds ratio, SD = standard deviation

^a^Metabolite concentrations with FDR < 0·001 were included in the table

^b^Metabolite concentrations were log-transformed and standardized (mean = 0, SD = 1).

^c^Odds ratio per 1-SD was generated from unconditional logistic regression models, adjusted for age at enrollment

^d^ Model was adjusted for age at baseline and *P. falciparum* status (positive and negative)

We observed statistically significant positive associations for BL with high ratio of cystine/cysteine (OR per 1-SD = 3·18, 95% CI 1·06, 9·52, *P* = 0·039) and low ratio of the sum of all lysophosphatidylcholines (LPCs) to the sum of all PCs (OR per 1-SD = 0·28, 95% CI 0·10, 0·78, *P* = 0·015, Table 3). Pathway and PC analyses showed significant positive associations of BL with nucleobase metabolites (OR = 15.60), dihydroceramides (OR = 4.36), and ceramides (OR = 2.79), as well as an inverse association of BL with LPCs (OR = 0.25) (Table 4).

**Table 3:** Association between metabolite ratios and Burkitt lymphoma based on 25 case-control pairs

Ratio of Metabolites	OR (95% CI) per 1 SD^a^	*P* value
Arginine/Ornithine	1·24 (0·54, 2·86)	0·61
GABR: Arginine / (Ornithine + Citrulline)	1·54 (0·63, 3·73)	0·34
Kynurenine/Tryptophan	1·60 (0·52, 4·95)	0·41
Ornithine/Citrulline	1·41 (0·55, 3·60)	0·47
Cystine/Cysteine	**3·18 (1·06, 9·52)**	**0·039**
Serine/Glycine	0·44 (0·18, 1·05)	0·064
Phenylalanine/Tyrosine	1·57 (0·55, 4·53)	0·40
Spermidine/Tryptophan	2·26 (0·73, 7·04)	0·16
DCA/CA	0·41 (0·16, 1·05)	0·063
BCA/Aromatic Amino Acids	1·45 (0·65, 3·26)	0·37
Sum of all LPCs divided by all PCs	**0·28 (0·1, 0·78)**	**0·015**

Abbreviations: OR = odds ratio, CI = confidence interval; LPC = lysophosphatidylcholines; PC = phosphatidylcholines

^a^ Models were adjusted for age at baseline, *P. falciparum status* (positive and negative), and hemoglobin concentration (low versus high, based on the mean concentration)

Aromatic acids are the sum of phenylalanine, tryptophan, and tyrosine

**Table 4:** Pathway analysis and principal components analysis (PCA) for the association between chemical sub-classes of plasma metabolites and Burkitt lymphoma in 25 case-control pairs ^a^

Chemical Sub-Class Pathway	Pathway analysis		PCA analysis for pattern score ^b^	
	No. of contributing metabolites	*P*-value	OR (95% CI), per 1 SD	*P*-value
Significant in PCA				
Nucleobases and related compounds	2	< 0.0001	15.60 (2.25, 108.16)	0.0054
Dihydroceramides	4	0.012	4.36 (1.43, 13.26)	0.0095
Lysophosphatidylcholines	9	0.002	0.25 (0.082, 0.76)	0.014
Ceramides	23	0.001	2.79 (1.04, 7.48)	0.042
Not significant in PCA^c^				
Acylcarnitines	2	< 0.0001	3.69 (0.91, 15.02)	0.068
Diglycerides	15	< 0.0001	2.63 (0.87, 7.93)	0.086
Indoles and derivatives	3	0.001	1.47 (0.59, 3.62)	0.41
Biogenic amines	5	0.001	2.52 (0.87, 7.28)	0.088
Dihexosylceramides	9	0.001	2.17 (0.86, 5.51)	0.10
Sphingomyelins	14	0.001	2.00 (0.77, 5.15)	0.15
Triglycerides	173	0.001	2.58 (0.87, 7.64)	0.086
Amino acid related	20	0.006	1.37 (0.55, 3.42)	0.50
Bile acids	13	0.02	2.20 (0.74, 6.49)	0.15
Hexosylceramides	17	0.02	2.08 (0.87, 4.99)	0.099
Phosphatidylcholines	69	0.04	1.09 (0.48, 2.46)	0.84
Trihexosylceramides	5	0.042	1.37 (0.59, 3.18)	0.46
Fatty acids	7	0.049	1.20 (0.51, 2.85)	0.68

Abbreviations: OR = odds ratio, CI = confidence interval

^a^ Pathway analysis: We examined the association between 17 chemical sub-classes of serum metabolites and risk of Burkitt lymphoma and tested a single *P*-value for pathways using a parametric bootstrap method. Within each bootstrap replication, *P*-values were generated from a vector of score test statistics from an estimated covariance matrix with a multivariate normal distribution (mean = 0). The pathway *P*-values are calculated based on 100,000 permutations. A Bonferroni correction was based on 17 classes with complete data with a *P*-value threshold = 0·0029 (0·05/17) considered significant

^b^ Models were adjusted for age at baseline, *P. falciparum* status (positive and negative) and hemoglobin concentration (low versus high)

^c^ Principal component analysis did not show a significant association for these pathways

## Discussion

Our findings demonstrate the ability to detect and quantify many plasma metabolites in children with and without BL in Uganda. We found that two metabolites, AABA and TAG (18:0_38:6), whose levels were detected mostly in BL cases, performed reliably to discriminate BL cases from controls in our study. Here we show that concentrations of these two plasma metabolites, plus *P. falciparum* infection, age and hemoglobin concetration significantly improved the discrimination of BL cases from controls with an AUC of 0.97, compared to an AUC of 0.85 when using only *P. falciparum* infection, age, and hemoglobin concetration. The AUC of 0.97 also is also higher than that obtained using plasma anti-EBV antibodies (AUC = 0.76) (Coghill et al., 2020), but comparable to that in studies that used EBV DNA copy number (AUC = 0.94) (Mulama et al., 2014; Westmoreland et al., 2017; Xian et al., 2021). Our results suggest that blood-based metabolites could provide another laboratory approach to facilitate the diagnosis of BL, which has hitherto only been shown in mice (Yang et al., 2017).

Our analysis is preliminary with notable limitations: the cross-sectional design limits temporal inferences; the sample size is small; we studied only boys; we performed multiple comparisons. The initial study design aimed to examine associations between environmental and endogenous risk factors for BL. However, our scope encompasses the investigation of the metabolomics profiles of BL, given that both environmental and endogenous factors that have been associated with disruptions in metabolite levels. While, the lack of prospective study to validate our findings, lack of data on comorbidities, and lack of data on diet are limitations, this was meant as a pilot study to test the feasibility of measuring metabolites in our sample set and to generate new hypotheses about metabolite associations with BL. Cross-sectional metabolite profiles give a broad “snapshot” view of differences in the body’s metabolism, which our pilot study suggests warrant closer study to examine the pathophysiology and risk profile of BL. AABA has been associated with early colorectal cancer (Ni et al., 2014), for example, and is a key intermediate in the synthesis of ophthalmic acid, a tripeptide analog of glutathione (Kar et al., 2017). It is possible that endogenous increases in AABA might be a biomarker of ophthalmic acid and associated oxidative damage due to altered cellular redox balance (Soga et al., 2006). Such a mechanism is consistent with our finding for the results assessed by the cystine/cysteine ratio (Banjac et al., 2008). However, increased AABA concentrations can also result from exogenous factors, particularly a diet rich in thiamin and vitamins B2, B3, and B6, as suggested in murine dietary studies (Kar et al., 2017). Diets rich in polyphenols may affect metabolite profiles by altering gut microbiota to favor *Clostridum sporogenes* (Gao et al., 2020), which can metabolize tryptophan to indole 3-propionic acid (IPA) (Peron et al., 2022). In our study, IPA showed a trend towards decreased ORs for BL (Supplementary Table 4), consistent with its reported anti-inflammatory and anti-oxidant activity (Negatu et al., 2020). In fact, IPA supplementation has been linked to cytostatic and anti-neoplastic properties in breast cancer (Sari et al., 2020).

Putrescine was associated with an increased OR for BL and is consistent with the role of deregulated polyamine metabolism in the development of cancer at all stages (Palmer, & Wallace, 2010). Decreasing intracellular concentrations of polyamines can produce cell stasis and/or cell death; ornithine decarboxylase, an enzyme that converts ornithine to putrescine, could be targeted therapeutically as it is upregulated in several cancers. Moreover, α-difluoromethylornithine (DFMO) (eflornithine), an irreversible inhibitor to ornithine decarboxylase, has been shown to be effective in primary prevention of colorectal cancer (Palmer, & Wallace, 2010). However, cancer cells can compensate for a decreased in polyamine synthesis by increasing uptake of exogenous polyamines via their polyamine transport system (PTS), including sodium dependent transporters for increasing uptake of putrescine (Palmer, & Wallace, 2010).

The high ratio of the sum of LPCs/the sum of PCs is considered a surrogate of phospholipase A2 (PLA2) activity (Park et al., 2012). PLA2 activity is typically associated with intravascular inflammation in cardiovascular disease (Lavi et al., 2008), as well as with brain swelling in cerebral malaria (Pappa et al., 2015). The inverse association with BL, which is opposite that with acute malaria, suggests that the pathophysiology of malaria in BL is different from that in acute malaria syndromes (Vadas et al., 1993). BL is associated with dysregulation of *MYC*, which is known to transactivate many genes, including of genes involved in fatty acid biosynthesis (Morrish et al., 2010); thus, associations with lipids could reflect reverse-causality. However, given that plasma lipids are elevated in people infected with *P. falciparum* (Faucher et al., 2002), and with recent reports indicating that lipids reprogram the Krebs cycle to become the preferred source of carbon (versus glucose or glutamine) and increases histone H3 and H4 acetylation and gene activation (McDonnell et al., 2016), it is possible that altered lipids may influence chromatin access in B cells with *IG*::*MYC* translocations (McDonnell et al., 2016) and contribute to progression to BL (Basso & Dalla-Favera, 2015; Robbiani et al., 2015). The inverse association between homoarginine, which is a competitive substrate for nitric oxide synthase (NO), with BL status is consistent with NO modulated transcriptional activity of the c-*MYC* gene promoter (Park, & Wei, 2003).

## Conclusion

In conclusion, we present preliminary results indicating that plasma metabolites are altered in children with BL compared to healthy controls, with concentrations of two metabolites TAG (18:0_38:6) and AABA as potential biomarkers of the presence of BL in children in Uganda. The findings warrant further examination, given the potential utility of differential abundance of metabolites to reduce diagnostic delays of BL in SSA.

### Electronic supplementary material

Below is the link to the electronic supplementary material.

Supplementary Material 1

## Data Availability

Access to covariate data for EMBLEM and Malawi studies can be requested directly from the corresponding author. The study forms are available for download from the EMBLEM website at: https://emblem.cancer.gov/resources/index.html.

## References

[CR1] Abdrabou, W., Dieng, M. M., Diawara, A., Serme, S. S., Almojil, D., Sombie, S., Henry, N. B., Kargougou, D., Manikandan, V., Soulama, I., & Idaghdour, Y. (2021). Metabolome modulation of the host adaptive immunity in human malaria. *Nat Metab*, *3*, 1001–1016. 10.1038/s42255-021-00404-9 34113019 10.1038/s42255-021-00404-9

[CR2] Alaggio, R., Amador, C., Anagnostopoulos, I., Attygalle, A. D., Araujo, I. B. O., Berti, E., Bhagat, G., Borges, A. M., Boyer, D., Calaminici, M., Chadburn, A., Chan, J. K. C., Cheuk, W., Chng, W. J., Choi, J. K., Chuang, S. S., Coupland, S. E., Czader, M., Dave, S. S., et al. (2022). The 5th edition of the World Health Organization Classification of Haematolymphoid Tumours: Lymphoid neoplasms. *Leukemia*, *36*, 1720–1748. 10.1038/s41375-022-01620-2 35732829 10.1038/s41375-022-01620-2 PMC9214472

[CR3] Ambrosio, M. R., Piccaluga, P. P., Ponzoni, M., Rocca, B. J., Malagnino, V., Onorati, M., De Falco, G., Calbi, V., Ogwang, M., Naresh, K. N., Pileri, S. A., Doglioni, C., Leoncini, L., & Lazzi, S. (2012). The alteration of lipid metabolism in Burkitt lymphoma identifies a novel marker: Adipophilin. *PLoS One*, *7*, e44315. 10.1371/journal.pone.0044315 22952953 10.1371/journal.pone.0044315 PMC3432109

[CR4] Andrade, C. M., Fleckenstein, H., Thomson-Luque, R., Doumbo, S., Lima, N. F., Anderson, C., Hibbert, J., Hopp, C. S., Tran, T. M., Li, S., Niangaly, M., Cisse, H., Doumtabe, D., Skinner, J., Sturdevant, D., Ricklefs, S., Virtaneva, K., Asghar, M., Homann, M. V., et al. (2020). Increased circulation time of Plasmodium Falciparum underlies persistent asymptomatic infection in the dry season. *Nature Medicine*, *26*, 1929–1940. 10.1038/s41591-020-1084-0 33106664 10.1038/s41591-020-1084-0

[CR5] Banjac, A., Perisic, T., Sato, H., Seiler, A., Bannai, S., Weiss, N., Kölle, P., Tschoep, K., Issels, R. D., Daniel, P. T., Conrad, M., & Bornkamm, G. W. (2008). The cystine/cysteine cycle: A redox cycle regulating susceptibility versus resistance to cell death. *Oncogene*, *27*, 1618–1628. 10.1038/sj.onc.1210796 17828297 10.1038/sj.onc.1210796

[CR6] Basso, K., & Dalla-Favera, R. (2015). Germinal centres and B cell lymphomagenesis. *Nature Reviews Immunology*, *15*, 172–184. 10.1038/nri3814 25712152 10.1038/nri3814

[CR7] Bornkamm, G. W. (2009). Epstein-Barr virus and the pathogenesis of Burkitt’s lymphoma: More questions than answers. *International Journal of Cancer*, *124*, 1745–1755. 10.1002/ijc.24223 19165855 10.1002/ijc.24223

[CR8] Broen, K., Dickens, J., Trangucci, R., Ogwang, M. D., Tenge, C. N., Masalu, N., Reynolds, S. J., Kawira, E., Kerchan, P., Were, P. A., Kuremu, R. T., Wekesa, W. N., Kinyera, T., Otim, I., Legason, I. D., Nabalende, H., Buller, I. D., Ayers, L. W., Bhatia, K., et al. (2023). Burkitt lymphoma risk shows geographic and temporal associations with Plasmodium falciparum infections in Uganda, Tanzania, and Kenya. *Proc Natl Acad Sci U S A*, *120*, e2211055120. 10.1073/pnas.2211055120 36595676 10.1073/pnas.2211055120 PMC9926229

[CR9] Chamba, C., Mbulaiteye, S. M., Balandya, E., & Schuh, A. (2023). Clinical application of circulating cell-free lymphoma DNA for fast and precise diagnosis of Burkitt lymphoma: Precision Medicine for Sub Saharan Africa. *Cambridge Prisms: Precision Medicine*, 1–20. 10.1017/pcm.2023.1 10.1017/pcm.2023.1 PMC10953760 38550928

[CR10] Coghill, A. E., Proietti, C., Liu, Z., Krause, L., Bethony, J., Prokunina-Olsson, L., Obajemu, A., Nkrumah, F., Biggar, R. J., Bhatia, K., Hildesheim, A., Doolan, D. L., & Mbulaiteye, S. M. (2020). The Association between the Comprehensive Epstein-Barr Virus Serologic Profile and endemic Burkitt Lymphoma. *Cancer Epidemiology, Biomarkers & Prevention*, *29*, 57–62. 10.1158/1055-9965.EPI-19-0551 10.1158/1055-9965.EPI-19-0551 PMC6954331 31619404

[CR11] Derkach, A., Otim, I., Pfeiffer, R. M., Onabajo, O. O., Legason, I. D., Nabalende, H., Ogwang, M. D., Kerchan, P., Talisuna, A. O., Ayers, L. W., Reynolds, S. J., Nkrumah, F., Neequaye, J., Bhatia, K., Theander, T. G., Prokunina-Olsson, L., Turner, L., Goedert, J. J., Lavstsen, T., et al. (2019). Associations between IgG reactivity to Plasmodium Falciparum erythrocyte membrane protein 1 (PfEMP1) antigens and Burkitt lymphoma in Ghana and Uganda case-control studies. *EBioMedicine*, *39*, 358–368. 10.1016/j.ebiom.2018.12.020 30579868 10.1016/j.ebiom.2018.12.020 PMC6355394

[CR12] Donati, D., Espmark, E., Kironde, F., Mbidde, E. K., Kamya, M., Lundkvist, A., Wahlgren, M., Bejarano, M. T., & Falk, K. I. (2006). Clearance of circulating Epstein-Barr virus DNA in children with acute malaria after antimalaria treatment. *Journal of Infectious Diseases*, *193*, 971–977. 10.1086/500839 16518759 10.1086/500839

[CR13] Faucher, J. F., Ngou-Milama, E., Missinou, M. A., Ngomo, R., Kombila, M., & Kremsner, P. G. (2002). The impact of malaria on common lipid parameters. *Parasitology Research*, *88*, 1040–1043. 10.1007/s00436-002-0712-6 12444452 10.1007/s00436-002-0712-6

[CR14] Gao, K., Mu, C. L., Farzi, A., & Zhu, W. Y. (2020). Tryptophan metabolism: A link between the gut microbiota and brain. *Adv Nutr*, *11*, 709–723. 10.1093/advances/nmz127 31825083 10.1093/advances/nmz127 PMC7231603

[CR15] Kar, S. K., Jansman, A. J. M., Schokker, D., Kruijt, L., Harms, A. C., Wells, J. M., & Smits, M. A. (2017). Amine Metabolism is influenced by Dietary protein source. *Front Nutr*, *4*, 41. 10.3389/fnut.2017.00041 28920057 10.3389/fnut.2017.00041 PMC5585152

[CR16] Lavi, S., Herrmann, J., Lavi, R., McConnell, J. P., Lerman, L. O., & Lerman, A. (2008). Role of lipoprotein-associated phospholipase A2 in atherosclerosis. *Current Atherosclerosis Reports*, *10*, 230–235. 10.1007/s11883-008-0036-9 18489851 10.1007/s11883-008-0036-9

[CR17] Legason, I. D., Pfeiffer, R. M., Udquim, K. I., Bergen, A. W., Gouveia, M. H., Kirimunda, S., Otim, I., Karlins, E., Kerchan, P., Nabalende, H., Bayanjargal, A., Emmanuel, B., Kagwa, P., Talisuna, A. O., Bhatia, K., Yeager, M., Biggar, R. J., Ayers, L. W., Reynolds, S. J., et al. (2017). Evaluating the Causal Link between Malaria Infection and endemic Burkitt Lymphoma in Northern Uganda: A mendelian randomization study. *EBioMedicine*, *25*, 58–65. 10.1016/j.ebiom.2017.09.037 29033373 10.1016/j.ebiom.2017.09.037 PMC5704046

[CR18] Li, Y., Webster-Cyriaque, J., Tomlinson, C. C., Yohe, M., & Kenney, S. (2004). Fatty acid synthase expression is induced by the Epstein-Barr virus immediate-early protein BRLF1 and is required for lytic viral gene expression. *Journal of Virology*, *78*, 4197–4206. 10.1128/jvi.78.8.4197-4206.2004 15047835 10.1128/jvi.78.8.4197-4206.2004 PMC374282

[CR19] López, C., Burkhardt, B., Chan, J. K. C., Leoncini, L., Mbulaiteye, S. M., Ogwang, M. D., Orem, J., Rochford, R., Roschewski, M., & Siebert, R. (2022). Burkitt lymphoma. *Nat Rev Dis Primers*, *8*, 78. 10.1038/s41572-022-00404-3 36522349 10.1038/s41572-022-00404-3

[CR20] Maziarz, M., Kinyera, T., Otim, I., Kagwa, P., Nabalende, H., Legason, I. D., Ogwang, M. D., Kirimunda, S., Emmanuel, B., Reynolds, S. J., Kerchan, P., Joloba, M. M., Bergen, A. W., Bhatia, K., Talisuna, A. O., Biggar, R. J., Goedert, J. J., Pfeiffer, R. M., & Mbulaiteye, S. M. (2017). Age and geographic patterns of Plasmodium Falciparum malaria infection in a representative sample of children living in Burkitt lymphoma-endemic areas of northern Uganda. *Malaria Journal*, *16*, 124. 10.1186/s12936-017-1778-z 28320389 10.1186/s12936-017-1778-z PMC5360076

[CR21] McDonnell, E., Crown, S. B., Fox, D. B., Kitir, B., Ilkayeva, O. R., Olsen, C. A., Grimsrud, P. A., & Hirschey, M. D. (2016). Lipids reprogram metabolism to become a Major Carbon Source for Histone Acetylation. *Cell Rep*, *17*, 1463–1472. 10.1016/j.celrep.2016.10.012 27806287 10.1016/j.celrep.2016.10.012 PMC5123807

[CR22] Moaddel, R., Zanos, P., Farmer, C. A., Kadriu, B., Morris, P. J., Lovett, J., Acevedo-Diaz, E. E., Cavanaugh, G. W., Yuan, P., Yavi, M., Thomas, C. J., Park, L. T., Ferrucci, L., Gould, T. D., & ZarateJr., C. A. (2022). Comparative metabolomic analysis in plasma and cerebrospinal fluid of humans and in plasma and brain of mice following antidepressant-dose ketamine administration. *Transl Psychiatry*, *12*, 179. 10.1038/s41398-022-01941-x 35501309 10.1038/s41398-022-01941-x PMC9061764

[CR23] Morrish, F., Noonan, J., Perez-Olsen, C., Gafken, P. R., Fitzgibbon, M., Kelleher, J., VanGilst, M., & Hockenbery, D. (2010). Myc-dependent mitochondrial generation of acetyl-CoA contributes to fatty acid biosynthesis and histone acetylation during cell cycle entry. *Journal of Biological Chemistry*, *285*, 36267–36274. 10.1074/jbc.M110.141606 20813845 10.1074/jbc.M110.141606 PMC2978554

[CR24] Mulama, D. H., Bailey, J. A., Foley, J., Chelimo, K., Ouma, C., Jura, W. G., Otieno, J., Vulule, J., & Moormann, A. M. (2014). Sickle cell trait is not associated with endemic Burkitt lymphoma: An ethnicity and malaria endemicity-matched case-control study suggests factors controlling EBV may serve as a predictive biomarker for this pediatric cancer. *International Journal of Cancer*, *134*, 645–653. 10.1002/ijc.28378 23832374 10.1002/ijc.28378 PMC3830732

[CR25] Na, J., Khan, A., Kim, J. K., Wadood, A., Choe, Y. L., Walker, D. I., Jones, D. P., Lim, C. S., & Park, Y. H. (2019). Discovery of metabolic alterations in the serum of patients infected with Plasmodium spp. by high-resolution metabolomics. *Metabolomics*, *16*, 9. 10.1007/s11306-019-1630-2 31872321 10.1007/s11306-019-1630-2

[CR26] Negatu, D. A., Gengenbacher, M., Dartois, V., & Dick, T. (2020). Indole Propionic Acid, an unusual antibiotic produced by the gut microbiota, with anti-inflammatory and antioxidant properties. *Frontiers in Microbiology*, *11*, 575586. 10.3389/fmicb.2020.575586 33193190 10.3389/fmicb.2020.575586 PMC7652848

[CR27] Ni, Y., Xie, G., & Jia, W. (2014). Metabonomics of human colorectal cancer: New approaches for early diagnosis and biomarker discovery. *Journal of Proteome Research*, *13*, 3857–3870. 10.1021/pr500443c 25105552 10.1021/pr500443c

[CR28] Ozuah, N. W., Lubega, J., Allen, C. E., & El-Mallawany, N. K. (2020). Five decades of low intensity and low survival: Adapting intensified regimens to cure pediatric Burkitt lymphoma in Africa. *Blood Adv*, *4*, 4007–4019. 10.1182/bloodadvances.2020002178 32841337 10.1182/bloodadvances.2020002178 PMC7448606

[CR29] Palmer, A. J., & Wallace, H. M. (2010). The polyamine transport system as a target for anticancer drug development. *Amino Acids*, *38*, 415–422. 10.1007/s00726-009-0400-2 19956998 10.1007/s00726-009-0400-2

[CR30] Pappa, V., Seydel, K., Gupta, S., Feintuch, C. M., Potchen, M. J., Kampondeni, S., Goldman-Yassen, A., Veenstra, M., Lopez, L., Kim, R. S., Berman, J. W., Taylor, T., & Daily, J. P. (2015). Lipid metabolites of the phospholipase A2 pathway and inflammatory cytokines are associated with brain volume in paediatric cerebral malaria. *Malaria Journal*, *14*, 513. 10.1186/s12936-015-1036-1 26691993 10.1186/s12936-015-1036-1 PMC4687364

[CR32] Park, S. W., & Wei, L. N. (2003). Regulation of c-myc gene by nitric oxide via inactivating NF-kappa B complex in P19 mouse embryonal carcinoma cells. *Journal of Biological Chemistry*, *278*, 29776–29782. 10.1074/jbc.M303306200 12783888 10.1074/jbc.M303306200

[CR31] Park, J. B., Lee, C. S., Jang, J. H., Ghim, J., Kim, Y. J., You, S., Hwang, D., Suh, P. G., & Ryu, S. H. (2012). Phospholipase signalling networks in cancer. *Nature Reviews Cancer*, *12*, 782–792. 10.1038/nrc3379 23076158 10.1038/nrc3379

[CR33] Peprah, S., Ogwang, M. D., Kerchan, P., Reynolds, S. J., Tenge, C. N., Were, P. A., Kuremu, R. T., Wekesa, W. N., Sumba, P. O., Masalu, N., Kawira, E., Magatti, J., Kinyera, T., Otim, I., Legason, I. D., Nabalende, H., Dhudha, H., Ally, H., Genga, I. O., et al. (2020). Risk factors for Burkitt lymphoma in east African children and minors: A case-control study in malaria-endemic regions in Uganda, Tanzania and Kenya. *International Journal of Cancer*, *146*, 953–969. 10.1002/ijc.32390 31054214 10.1002/ijc.32390 PMC6829037

[CR34] Peron, G., Merono, T., Gargari, G., Hidalgo-Liberona, N., Minarro, A., Lozano, E. V., Castellano-Escuder, P., Gonzalez-Dominguez, R., Del Bo, C., Bernardi, S., Kroon, P. A., Cherubini, A., Riso, P., Guglielmetti, S., & Andres-Lacueva, C. (2022). A Polyphenol-Rich Diet increases the gut microbiota metabolite indole 3-Propionic Acid in older adults with preserved kidney function. *Molecular Nutrition & Food Research*, *66*, e2100349. 10.1002/mnfr.202100349 35315592 10.1002/mnfr.202100349 PMC9787513

[CR35] Robbiani, D. F., Deroubaix, S., Feldhahn, N., Oliveira, T. Y., Callen, E., Wang, Q., Jankovic, M., Silva, I. T., Rommel, P. C., Bosque, D., Eisenreich, T., Nussenzweig, A., & Nussenzweig, M. C. (2015). Plasmodium infection promotes genomic instability and AID-Dependent B cell lymphoma. *Cell*, *162*, 727–737. 10.1016/j.cell.2015.07.019 26276629 10.1016/j.cell.2015.07.019 PMC4538708

[CR36] Sari, Z., Miko, E., Kovacs, T., Janko, L., Csonka, T., Lente, G., Sebo, E., Toth, J., Toth, D., Arkosy, P., Boratko, A., Ujlaki, G., Torok, M., Kovacs, I., Szabo, J., Kiss, B., Mehes, G., Goedert, J. J., & Bai, P. (2020). Indolepropionic Acid, a metabolite of the Microbiome, has Cytostatic properties in breast Cancer by activating AHR and PXR receptors and inducing oxidative stress. *Cancers (Basel)*, *12*. 10.3390/cancers12092411 10.3390/cancers12092411 PMC7565149 32854297

[CR37] Soga, T., Baran, R., Suematsu, M., Ueno, Y., Ikeda, S., Sakurakawa, T., Kakazu, Y., Ishikawa, T., Robert, M., Nishioka, T., & Tomita, M. (2006). Differential metabolomics reveals ophthalmic acid as an oxidative stress biomarker indicating hepatic glutathione consumption. *Journal of Biological Chemistry*, *281*, 16768–16776. 10.1074/jbc.M601876200 16608839 10.1074/jbc.M601876200

[CR38] Vadas, P., Taylor, T. E., Chimsuku, L., Goldring, D., Stefanski, E., Pruzanski, W., & Molyneux, M. E. (1993). Increased serum phospholipase A2 activity in Malawian children with falciparum malaria. *American Journal of Tropical Medicine and Hygeine*, *49*, 455–459. 10.4269/ajtmh.1993.49.455 10.4269/ajtmh.1993.49.455 8214274

[CR39] Weiss, D. J., Lucas, T. C. D., Nguyen, M., Nandi, A. K., Bisanzio, D., Battle, K. E., Cameron, E., Twohig, K. A., Pfeffer, D. A., Rozier, J. A., Gibson, H. S., Rao, P. C., Casey, D., Bertozzi-Villa, A., Collins, E. L., Dalrymple, U., Gray, N., Harris, J. R., Howes, R. E., et al. (2019). Mapping the global prevalence, incidence, and mortality of Plasmodium Falciparum, 2000-17: A spatial and temporal modelling study. *Lancet*, *394*, 322–331. 10.1016/s0140-6736(19)31097-9 31229234 10.1016/s0140-6736(19)31097-9 PMC6675740

[CR40] Westmoreland, K. D., Montgomery, N. D., Stanley, C. C., El-Mallawany, N. K., Wasswa, P., van der Gronde, T., Mtete, I., Butia, M., Itimu, S., Chasela, M., Mtunda, M., Kampani, C., Liomba, N. G., Tomoka, T., Dhungel, B. M., Sanders, M. K., Krysiak, R., Kazembe, P., Dittmer, D. P., et al. (2017). Plasma Epstein-Barr virus DNA for pediatric Burkitt lymphoma diagnosis, prognosis and response assessment in Malawi. *International Journal of Cancer*, *140*, 2509–2516. 10.1002/ijc.30682 28268254 10.1002/ijc.30682 PMC5386821

[CR41] White, N. J. (2018). Anaemia and malaria. *Malaria Journal*, *17*, 371. 10.1186/s12936-018-2509-9 30340592 10.1186/s12936-018-2509-9 PMC6194647

[CR42] Xian, R. R., Kinyera, T., Otim, I., Sampson, J. N., Nabalende, H., Legason, I. D., Stone, J., Ogwang, M. D., Reynolds, S. J., Kerchan, P., Bhatia, K., Goedert, J. J., Mbulaiteye, S. M., & Ambinder, R. F. (2021). Plasma EBV DNA: A promising diagnostic marker for endemic Burkitt Lymphoma. *Frontiers in Oncology*, *11*, 804083. 10.3389/fonc.2021.804083 34970500 10.3389/fonc.2021.804083 PMC8713969

[CR43] Yang, F., Du, J., Zhang, H., Ruan, G., Xiang, J., Wang, L., Sun, H., Guan, A., Shen, G., Liu, Y., Guo, X., Li, Q., & Tang, Y. (2017). Serum metabolomics of Burkitt Lymphoma Mouse Models. *PLoS One*, *12*, e0170896. 10.1371/journal.pone.0170896 28129369 10.1371/journal.pone.0170896 PMC5271368

